# Radiation therapy for pancreatic adenocarcinoma, a treatment option that must be considered in the management of a devastating malignancy

**DOI:** 10.1186/s13014-019-1277-1

**Published:** 2019-06-26

**Authors:** William A. Hall, Karyn A. Goodman

**Affiliations:** 1 Department of Radiation Oncology, Medical College of Wisconsin, Wauwatosa, USA; 2 Department of Radiation Oncology, University of Colorado, Aurora, CO USA

**Keywords:** Pancreatic cancer, Pancreatic adenocarcinoma, Pancreatic radiation therapy, Pancreatic SBRT, PRODIGE trial, PREOPANC-1 trial

## Abstract

Clinical outcomes for patients with pancreatic adenocarcinoma (PAC) remain dismal. Local recurrences, proportions of margin positive surgical resections, and overall survival outcomes remain inferior in PAC than any other solid tumor. This stems from a current standard of care management approach that needs to be inspired and transformed with modern treatment techniques and novel therapeutic options. Radiation therapy has historically been a central component in the treatment of pancreatic adenocarcinoma; however, the role of radiation therapy has been called into question based on the publication of clinical trials with conflicting results. We present an overview of the rationale for radiation therapy in resectable, borderline resectable, and unresectable pancreatic adenocarcinoma. We further present a summary of emerging clinical data and future directions to improve outcomes in this devastating malignancy.

## Background

Pancreatic adenocarcinoma (PAC) remains one of the single most devastating malignancies in existence. Median overall survival remains dismal for the vast majority of patients afflicted with PAC. It has risen to the fourth leading cause of cancer death in the United States (US) [1]. Moreover, in the next fifteen years, the projected impact of PAC is expected to increase, placing it as one of the top three causes of cancer death by 2030 [2]. The reasons for this are multifactorial. One of the dominant factors is that only a minority of patients with PAC fall into the most favorable category of surgically resectable, without clinical evidence of metastatic disease. Yet even for patients with resectable disease, the only subgroup with a potential for cure, oncologic outcomes remain abysmal. The presence of such poor outcomes requires careful consideration and examination of current management approaches to this malignancy. Current strategies for PAC, even for patients with the best prognosis, are simply not working.

Treatment for PAC has been the subject of controversy for decades. Despite, and often because of, the results of multiple contradictory clinical trials that have examined various management strategies, oncologists remain confused, conflicted, and opinionated as to the optimal manner in which PAC should be treated. At the center of much of this controversy is radiation therapy. In this article we attempt to articulate the changes to radiation therapy that have taken place over the past decade, to critically review the current literature assessing the use of radiation therapy in older trials, to present comparative data supporting its use, and we advocate for continued close examination of radiation therapy by oncologists for the management of PAC.

## Main text

### Evolution of radiation therapy and its critical relationship with the therapeutic index in PAC

Radiation therapy represents a highly complex and technical treatment that is rapidly evolving. The modality has undergone, and continues to undergo, dramatic transformations with advances in computational modeling and medical imaging. These advances will only continue to accelerate in the coming years, and will likely follow a “double exponential” growth pattern leveraging advances in both hardware and software capabilities. While radiation therapy has historically been described as a singular category in oncologic management, in reality, it represents a broad treatment class, inclusive of a wide range of treatment procedures and methods. Despite the spectrum of potential treatments under the category of radiation therapy, the all-encompassing nomenclature often leaves other oncologic specialties confused as to the heterogeneity that exists within the term radiation therapy. This leads radiation oncologist to ask numerous questions: was the entire tumor treated to the prescription dose, what about regional lymph nodes, what type of daily imaging was used to align the patient, what normal structure tolerances and variations did the planning allow, what were the margins used, and treatment modality? While this type of variability may seem purely academic, it has critical implications on endpoints, such as overall survival. In one of the first examinations of the importance of protocol specified radiation guidelines in PAC, Abrams et al., presented that deviations from protocol specified guidelines significantly impacted the overall survival of patients treated on RTOG 9704 [3]. These variations were despite clear guidelines in the RTOG 9704 parent protocol as to how radiation oncologists should be applying radiation therapy. The critical influence that even subtle deviations can have on survival outcomes for patients treated with radiation therapy requires careful attention. Such a significant impact highlights the narrow therapeutic index associated with the use of radiation therapy in PAC. When radiation oncologists dismiss or criticize poor clinical trial results secondary to an absence of quality assurance, radiation compliance data, or central review of treatment plans, it stems from the understanding that even minor deviations in plan quality can substantially impact patient outcomes in this malignancy. As radiation therapy complexity exponentially improves with advances in delivery methods this type of data will become increasingly important for future clinical trials.

### The role of radiation therapy in resectable PAC

When considering the most favorable patients with PAC the outcomes should be excellent, unfortunately they remain dismal. Currently, the role of radiation therapy is controversial in patients with resectable PAC due to the lack of definitive data evaluating the use of adjuvant therapy using modern radiotherapy techniques. The initial study evaluating adjuvant chemotherapy and radiation for PAC, the Gastrointestinal Tumor Study Group (GITSG) clinical trial, established adjuvant radiotherapy to be superior to observation in patients with resected PAC [4]. The study was stopped early due to poor accrual (43 patients in 8 years). It showed, however, that the treated group experienced a survival benefit with longer median survival (21.0 months vs. 10.9 months; *p* < 0.05) and 2-year survival (43% vs. 19%). This study was criticized for its small sample size and low radiation dose (40Gy split course) [5]. Following the publication of the GITSG trial, the European Study Group for Pancreatic Cancer 1 (ESPAC-1) trial was conducted, this was a pivotal historic moment in the management of patients with PAC, particularly with regard to the role of radiation therapy. Notable is that this trial has been widely criticized, particularly with regard to the radiation therapy delivered [6, 7]. In this multi-center, 2 × 2 factorial design, 73 patients with resected pancreatic adenocarcinoma were assigned to chemoradiation therapy alone, 75 patients to chemotherapy alone, 72 patients to both chemoradiation therapy and chemotherapy, and 69 patients to observation. Again, the critiques of this study abound, particularly with regard to the radiation therapy [8]. The radiation delivered in this study was antiquated and a wide range of doses and techniques could have been employed. The critiques of the ESPAC-1 trial have been the subject of multiple editorials and a detailed review of these is beyond the scope of this article. In summary, it is well understood, by modern standards, that such radiation therapy doses and treatment strategies applied in either the GITSG trial or the ESPAC-1 are woefully inferior to contemporary radiation standards. The recommended radiation dose for the trial, 40 Gy delivered in a split course fashion with a break between the delivery of the first and second half of treatment, is clearly a biologically ineffective dose and delivery method. In fact, the split course has been shown to be inferior in many other cancer types, and may actually allow for accelerated repopulation of tumor cells that could lead to worse outcomes [9–11]. Judgements regarding the use of radiation therapy for patients with resectable pancreatic adenocarcinoma based on the ESPAC-1 trial should be made with extreme caution. Nevertheless, the publication of the ESPAC-1 trial has led to subsequent omission of radiation therapy from most European adjuvant trials, including ESPAC-3 [12] and ESPAC-4 [13]. In North America, radiation therapy has remained part of the adjuvant treatment strategy, and several trials have been presented since then, including RTOG 9704, however none have examined, in a randomized fashion, the role of modern era radiation therapy [14]. The successor trial to RTOG 9704, RTOG 0848, will help to answer the question regarding the role of adjuvant chemo-RT. RTOG 0848 has completed enrollment and results are anticipated in the coming years. Although the RTOG 0848 addresses the adjuvant radiotherapy question, the recent publication of the PRODIGE trial which demonstrated a significant benefit for adjuvant FOLFIRINOX over gemcitabine alone, has established FOLFIRINOX as the standard of care for adjuvant chemotherapy. Thus, the impact of adjuvant chemoradiation after gemcitabine-based chemotherapy may be less relevant. Nonetheless, local failure rates were still high on the PRODIGE study with a component of local/regional present in over 20% of cases [23]. To date the question as to the role of post-operative radiation in PAC, as addressed in a phase III trial, using modern era RT with robust quality assurance, remains unanswered.

### Neoadjuvant treatment in PAC

Highly perplexing in the management of PAC is the commitment of oncologists to the paradigm of upfront surgical resection. This treatment approach for patients described as “resectable” seems to defy nearly all other oncologic standards applied to other solid tumors throughout the body. Consider the ESPAC-4 clinical trial in which patients were randomized to two different chemotherapy schedules after treatment with upfront surgical resection. A staggering 60% of patients had surgical margins that were defined pathologically as “positive.” Tragically, half the patients on this trial went on to develop a local recurrence, which can often be a morbid and life threatening event [13, 15]. The presence of margin positivity in 60% of patients is simply unprecedented in any other extra-cranial solid tumor managed with upfront surgical resection. Even for these resectable patients, who represent the rarest and most favorable patients with PAC, the outcomes remained poor with a median overall survival of 28 months. Not surprisingly, patients with positive margins had particularly poor outcomes [15]. We challenge oncologists to identify a solid tumor elsewhere in the body with margin positivity rates of 60% and local recurrence rates of 50% that does not routinely undergo treatment with neoadjuvant therapy. Upfront surgical resection for PAC, even in those patients considered “surgically resectable”, should be given careful consideration as to the oncologic rationale supporting this treatment approach. It is in the neoadjuvant setting that radiation therapy likely has the most benefit given the intact tumor microvasculature and more favorable environment to radiation therapy induced cell kill. While cross trial comparisons are fraught with challenges, Table 1 compares pathologic outcomes for patients managed with neoadjuvant chemoradiation therapy as compared with upfront surgical resection followed by adjuvant chemotherapy.

**Table 1:** Summary Rationale For Neoadjuvant Chemo-RT Versus Adjuvant Chemo For Pancreatic Adenocarcinoma

Comparator Variable	Neoadjuvant Chemo-RT	Up-front Surgery + Adjuvant Chemo	Citations to Support
Rate of Positive Margins	2–20%	16–60%	[16–19]
Incidence of Node Positivity	17–40%	62–80%	[13, 16, 17, 20]
Successful Treatment completion	70–80%	50–60%	[17, 21, 22]
Rates of Local Recurrence	5–15%	19–53%	[13, 17, 18, 23]

The recently presented trial titled: *Preoperative chemoradiotherapy* versus *immediate surgery for resectable and borderline resectable pancreatic cancer: A Randomized, Controlled, multicenter, phase III trial (PREOPANC-1)* provided some additional insight on the potential importance of neoadjuvant therapy for PAC. This trial (not yet published in manuscript form) randomized 246 patients to immediate surgery (arm A-127 patients) as compared with preoperative chemoradiotherapy (arm B- 119 patients). Both of these arms were followed by adjuvant gemcitabine-based chemotherapy. While the results are preliminary, it appears that preoperative chemoradiotherapy demonstrated an improvement in overall survival with 13.7 months as compared with 17.1 months, *p*-value of 0.074 [24]. In addition, the R0 resection rate was significantly improved with neoadjuvant therapy from 31 to 63%, and disease free survival was also improved from 7.9 months to 9.9 months (*p* = 0.023). The final manuscript publication from this work is eagerly awaited, yet lends support to the role of neoadjuvant therapy. Additional randomized trials are needed in the neoadjuvant setting to better understand the role of radiation therapy, given neoadjuvantly, as compared with chemotherapy. Fortunately the ongoing ESPAC-5 trial is examining the role of various combinations of neoadjuvant therapy with upfront surgical resection. Additional trials are needed to explore variations in radiation therapy (including dose, fractionation, and treatment volumes) given pre-operatively for patients with PAC.

### Locally advanced pancreatic cancer

Locally advanced, or surgically unresectable, PAC remains one of the single most deadly malignancies in existence. Unfortunately, options for patients with unresectable PAC remain limited and relatively ineffective. Whenever possible, patients with locally advanced PAC should be treated in a clinical trial. When examining the role for radiation therapy, it seems increasingly clear that historic radiation treatment strategies, using conventionally fractionated radiation, need improvement. This was highlighted in the LAP-07 Phase III trial in which patients with locally advanced PAC were randomized after neoadjuvant chemotherapy to either continuation of treatment with chemotherapy or to treatment with concurrent chemoradiation [25]. The radiation therapy was to a total dose of 54 Gy over 30 fractions with concurrent capecitabine. This was given using 3D conformal radiation, and prophylactic regional nodal radiation was not included. Median overall survival was not improved by the addition of chemoradiation. Chemoradiation was associated with decreased local progression. The LAP-07 trial conflicted with the smaller Eastern Cooperative Oncology Group (ECOG) that did demonstrate an improvement in overall survival with the use of chemoradiation as compared with chemotherapy alone in patients with unresectable PAC [26]. These two trials seem to highlight the narrow therapeutic index associated with the use radiation therapy in patients with PAC, and also illustrate the failure of historic radiation techniques to yield any promising results for this devastating malignancy. With median overall survivals ranging from 9 to 13 months these trials further highlight the need for considerable investigation into manners by which outcomes can be improved in patients with locally advanced PAC. Novel radiation techniques, such as stereotactic body radiation therapy (SBRT), may offer a more convenient and possibly more biologically effective alternative to conventionally fractionated radiation therapy, however additional research is needed to understand the optimal dose and delivery strategy [27]. This is particularly the case as the overall survival in many modern SBRT clinical trials for PAC have not demonstrated marked improvement over historic fractionated chemo-RT trials, or recent publications of dose escalated radiation therapy [22, 28]. In addition, the potential for marginal miss, or local recurrence, is something that should be carefully considered when using SBRT for PAC. Given that approximately 30% of patients with locally advanced PAC die from isolated locally destructive pancreatic cancer it seems that improved patient selection could help to identify those patients optimally suited for local-regional therapy [29]. Novel methods to identify patients at particularly high risk for local recurrence are considerably needed.

### Future directions

Advanced methods of radiation therapy, such as real-time MR guidance, may improve the ability to target PAC while reducing radiation dose to the small bowel, thereby improving the therapeutic index of radiation therapy (PMID 30932367) [30]. It is imperative that radiation oncologists conduct high quality, prospective clinical research evaluating this novel technology to prove its efficacy. This technology presents tremendous potential advantages for PAC, but must be robustly evaluated and proven. As radiation therapy exponentially advances in the coming decade, the opportunities to improve outcomes in this devastating malignancy will only continue to increase.

## Conclusions

In order to create a more promising future for PAC patients there is a need for new clinical trials. Such trials should have a focus on neoadjuvant therapeutic strategies, novel radiation delivery techniques, and improved patient selection. Radiation therapy is rapidly improving, and is a highly precise modality that continues to offer great promise. Given the exceedingly high rates of local recurrence and margin positivity after surgical resection for PAC, radiation therapy must be given careful consideration as a critical modality for future consideration to improve outcomes in this devastating malignancy. Finally, as systemic therapy improves and patients are living longer, with better control of the distant metastases, local control of the primary site becomes more critical. Thus, the role of radiation therapy may continue to expand as an option for patients with unresectable disease and as neoadjuvant therapy in the borderline resectable and resectable PAC setting.

## Abbreviations

GITSG: Gastrointestinal Tumor Study Group
MR: Magnetic Resonance
PAC: Pancreatic adenocarcinoma
RTOG: Radiation Therapy Oncology Group
SBRT: Stereotactic Body Radiation Therapy
